# A258 SEVERE ACUTE HEPATITIS OF UNKNOWN ORIGIN WITH RAPID PROGRESSION TO PAEDIATRIC ACUTE LIVER FAILURE IN A CHILD

**DOI:** 10.1093/jcag/gwac036.258

**Published:** 2023-03-07

**Authors:** V Venkatesh, A Ghanekar, B Sayed, I Siddiqui, V Ng, M Miserachs

**Affiliations:** 1 Pediatric Gastroenterology, Hepatology and Nutrition; 2 Surgery; 3 Pathology, The Hospital for Sick Children, Toronto, Canada

## Abstract

**Background:**

Severe acute hepatitis (SAH) of unknown origin among children has been a major public health concern globally in recent times, with >1010 cases reported to WHO, including 28 in Canada as of July2022. The clinical syndrome in all identified cases (median age 3, IQR 2-5 yrs) was acute hepatitis with progression to paediatric acute liver failure (PALF) and need for liver transplantation (LT) in 5.4-13%. Human adenovirus (HAdV), particularly serotypes 40 and 41, remains the most common pathogen detected around the time of presentation in upto 70%. HAdV hepatitis is a rarity in the immunocompetent. The role of adenovirus in SAH remains unclear and investigations continue

**Purpose:**

To report the case of a child meeting the WHO case definition of SAH of unknown origin in whom adenovirus was detected with rapid progression to PALF requiring LT

**Method:**

Case report and literature review

**Result(s):**

A previously healthy 4year-old girl presented to us with a 1week history of abdominal pain and vomiting followed by onset of jaundice. 1month prior to presentation, she had an episode of conjunctivitis. Examination revealed a well-appearing girl with icterus, hepatosplenomegaly with no stigmata of chronic liver disease and no features of hepatic encephalopathy. Laboratory results on day of presentation revealed transaminases >10 times upper limit of normal (ALT 4686U/L, AST 5986U/L), total bilirubin-233μmol/L (conjugated bilirubin-153μmol/L) and INR-1.6. Work up for viral hepatitis(A-E), metabolic, autoimmune, genetic or mechanical causes of hepatitis was negative (Table 1). HAdV was detected by PCR in blood (9700 copies/mL), stool and nasopharyngeal swab. Treatment with cidofovir (1mg/kg/dose) was started on day8 after presentation.By day11, laboratory parameters had worsened with ALT 1293U/L, AST 2326U/L, total bilirubin 331μmol/L, INR-9.1 and LT was considered. With failure to improve over the next 48hrs, she received a living donor LT on day13 after presentation and had an uneventful post-transplant course. At the time of writing this report, she was 41 days post-transplant, doing well on immunosuppression with tacrolimus and tapering dose of steroids. Histopathological examination of liver showed extensive hepatocyte loss of upto 80%, replaced by ductules, in a background of mild hepatitis (patchy pan-lobular inflammation with minimal portal inflammation) and no significant fibrosis. Electron microscopy (EM) showed patchy hemophagocytosis. No evidence of HAdV on immunohistochemical stains or EM was identified. These findings are directly in line with what others have reported, namely a lack of direct toxic effect of virus on liver tissue

**Image:**

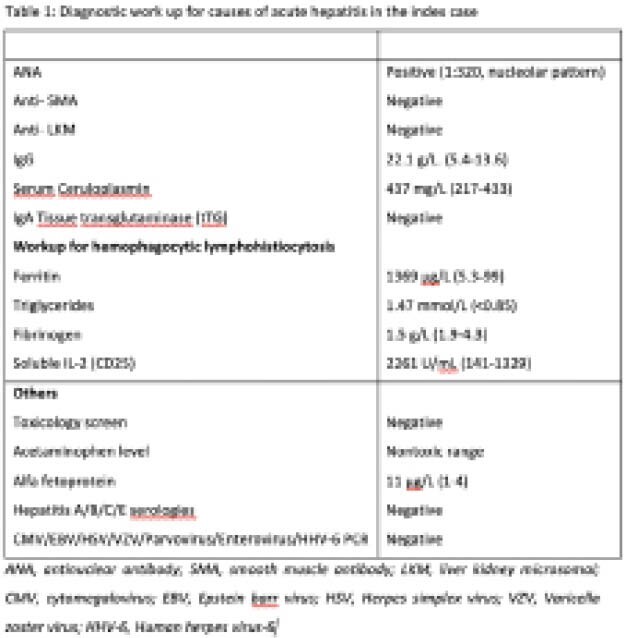

**Conclusion(s):**

This case highlights the potential for rapid progression to PALF and need for LT in a child SAH of unknown origin. Early identification and diagnosis of PALF is important and should be followed by transfer to a LT center. As previously described by others, HAdV was detected, but its role in pathogenesis of this clinical syndrome remains elusive

**Please acknowledge all funding agencies by checking the applicable boxes below:**

None

**Disclosure of Interest:**

None Declared

